# Supplementary material for: Women empowerment and sexually transmitted infections: Evidence from Bangladesh demographic and health survey 2014
Source: PLoS One. 2022 Feb 16;17(2):e0263958. doi: 10.1371/journal.pone.0263958 (PMC8849524; doi:10.1371/journal.pone.0263958)
Supplement: Attachment — Submitted filename: Responses to Reviewers.docx [file pone.0263958.s001.docx]

**Response to Editor Comments**

1. Line 113 mentioned that “All the independent variables are shown in Table 1”. It will be Table 2.

Response: Thank you. We have corrected it in line 144 of the unmarked manuscript file.

2. The authors have not constructed an empowerment scale/index. They used the indicators as a separate variable

Response: Thank you for highlighting this important concern, we appreciate it. It was possible to employ a women empowerment index, such as the Survey-based Women's Empowerment Index-^1^. However, rather than using a composite score, we were interested in investigating how different women empowerment indicators might affect women's self-reported abnormal genital discharge and genital sores differently^2^.

Reference:

1. Ewerling F, Lynch JW, Victora CG, van Eerdewijk A, Tyszler M, Barros AJ. The SWPER index for women's empowerment in Africa: development and validation of an index based on survey data. The Lancet Global Health. 2017 Sep 1;5(9):e916-23.

2. Mainuddin A, Begum HA, Rawal LB, Islam A, Islam SS. Women empowerment and its relation with health seeking behavior in Bangladesh. J Family Reprod Health. 2015;9(2):65.

3. The division has been written as ‘Province.’

Response: Thank you. In the context of Bangladesh, the term “province” is not officially used. The word “Division” is an officially accepted term. We have corrected it in Table 2, Line 148, 194, Table 3, Table 4 of the unmarked manuscript file (Table 2, Line 117, 145, 148)

4. The authors have presented a conceptual framework where knowledge about safe sexual behavior and treatment-seeking behavior for STI symptoms have been mentioned. However, I did not see any such variable in the list of explanatory variables and the results

Response: Thank you for your comment. Women's socio-demographic characteristics and indicators of women's empowerment have direct and indirect effects on abnormal genital discharge and genital sores. A single direct arrow indicates a direct effect. Women’s demographic characteristics and empowerment indicators indirectly also affect outcomes through safer sex knowledge and practices and health-seeking behaviour. Since we do not have data on safer sex knowledge, practices and health-seeking behaviour, our model exclusively focused on the direct effect. Now we have revised the statistical analysis section and explained it in Lines 158–169 of the unmarked manuscript file.

5. The reported sample 16858 does not match with the DHS report. The same goes for the proportion of women who reported having signs of genital sores and abnormal genital discharge. Please check the original report

Response: Many thanks for your comment. Our sample size was different from the BDHS sample size because the BDHS report [Table 3.1 (page 29) and Table 12.7 (page 184)] calculated the prevalence among ever-married women, which included married, widowed, divorced, and women who were no longer living together (sample size=17,868). However, we limited our analysis to currently married women and thus used a sample size of 16,858 women. We referenced it in the study design and sampling technique section, line 130-132 of the unmarked manuscript file.

6. In table 3, the authors have reported findings of the variable “Control over their ow earnings”. However, the ‘n’ is different from the total. However, no explanation has been provided.

Response: We appreciate your comment. In table 3, now we have included a footnote that as a result of missing values, the total may not equal 100.0 percent.

7. Line 162 mentioned, “Impact of women empowerment on abnormal genital discharge and genital sores”. Please consider revisiting the use of “impact” as the current study design does not allow the authors to see the impact.

Response: We appreciate this concern. We have revised it to the association between women empowerment indicators and abnormal genital discharge and genital sores in line 211-212 in the unmarked manuscript file.

**___________________________________________________________________________________**

**Response to Reviewer 1 Comments**

**On Abstract**

Comment 1: The second sentence of your abstract is not clear. What poses difficulties and what remains untreated? I think you should read it again and try to make it clear. It can be rephrased as … many women remain untreated

Response: We appreciate your suggestion. We used the term "difficulty" and "untreated" to refer to "challenges" and "undiagnosed." However, in the unmarked manuscript, we revised lines 29-33 in the previous edition and inserted them in line -23-27.

Comment 2: Please write the word cross-tab in full

Response: Thank you for the suggestion. It has been inserted in unmarked manuscript lines 28-32. In addition, we revised the materials and methods section to reflect the fact that we used multilevel mixed-effect logistic regression as suggested by reviewer 2.

Comment 3: Change the and in “All the analysis was adjusted for multistage sampling design and cluster weight.” to …. using cluster weight

Response: We appreciate the suggestion. In line 32 (lines 35-36 in the previous edition or version?) of the unmarked manuscript, we have inserted the phrase using cluster weight.

Comment 4: The sentence that presents multivariate results is not clear. Please read it again and present it clearly. I think it should be presented as Multivariate analysis revealed that women’s lower reporting of STIs symptoms was significantly…..

Response: Thanks for the suggestion. We re-read and revised lines 34–39 of the unmarked manuscript (lines 38-41 in the previous version) as per your recommendation.

Comment 5: The figures in the abstract should be presented with one decimal place.

Response: Thank you for highlighting this. We have rounded the figures only in the abstract section.

**On Manuscript**

Comment 6: Your introduction needs thorough editing. Many of the sentences lack joining words and the manuscript is full of grammatical errors.

Response: We appreciate this suggestion. We have re-read and edited the introduction section thoroughly line-by-line, attempting to correct grammatical and syntax errors throughout the manuscript.

Comment 7: You should have some literature that connects other selected variables to real studies. The introduction is silent about these connections yet they are also study vaiables.

Response: We appreciate your recommendation. We revised the introduction section extensively, attempted to connect the study variables, and added some literature from lower-middle income countries.

Comment 8: The first sentence of the introduction is not clear. Seems you are joining two parts that are disconnected.

Response: Many thanks. We have revised this according to your suggestion.

Comment 9: Line 62 misses … in developing countries.

Response: We appreciate your diligence in following up on this. In line 55-65 of the unmarked manuscript, we added some literature pertaining to developing countries.

Comment 10: Line 62 It has also been reported that there is higher HIV transmission among reproductive-age women

Response: Thank you, we have deleted line 62 after thoroughly rewriting the introduction.

Comment 11: Line 65 misses … and genital herpes

Response: We appreciate your feedback, and we have removed line 65 after a detailed revision of the introduction.

Comment 12: Lines 90-93 are confusing about the dataset used in this study. You state that the 2017 BDHS did not have STI/HIV data but then conclude that you used the 2017 BDHS. I think line 92 should read …, BDHS 2014 …

Response: Thank you for highlighting this. It was a typing error, and we apologize for this. Now we have corrected it in line 114-118 of the unmarked manuscript (line 90-94 in the previous edition)

Comment 13: Since you have two outcomes, the subsection in line 105 should be outcome variables

Response: Thank you for the suggestion. We have been split sentence and added a few sentences. The new edition lines ran from 136 to 140, replacing the previous edition line of 106-109.

Comment 14: The word “referent” in the multivariate table should be changed to “reference”

Response: Many thanks, we have modified this in the table as suggested.

Comment 15: In the discussion, the sentence on line 215 should end on independently otherwise, the addition renders it confusing.

Response: Thanks for your comment. The clause in line 215 currently ends separately, and a clarification has been provided ('Also' has been omitted from the following sentence). The line is 253 in the unmarked version.

Comment 16: Line 220-223 is very questionable for women of reproductive age. Is this in the context of your community. It may only apply to the teenagers and young women. If so, please indicate this clearly. Otherwise many of the women of reproductive age may not be under any guardians!

Response: We appreciate this suggestion. The statement was made in reference to teenage and young women, which we have addressed now (Line-295). The previous version has a line number of 223.

Comment 17: In the conclusion, I think the issue of joint decision making can also be facilitated by encouraging couple communication. If it is applicable in the Bangladesh Context, please include it.

Response: Thanks for the suggestion. We agree with this, and we revised the conclusion (Line-346 in unmarked manuscript) which was Line-299 & 300 in the previous version.

_____________________________________________________________________________________

**Response to Reviewer 2 Comments**

**Introduction**

Comment 1: Introductory section is very short. The authors urged that (P. 9, L. 60-62) abnormal genital discharge is a common occurrence caused mostly by Chlamydia trachomatis, Neisseria gonorrhoeae, Trichomonas vaginalis and is associated with a higher risk of HIV transmission precisely developing countries. Citing of some findings from developing countries which showed that having STIs in a woman increases HIV would increase the quality of the MS. Are those infections limited to HIV, or some other diseases like cancer?

Response: Thank you for discussing this important question. Chlamydia trachomatis, Neisseria gonorrhoeae, Trichomonas vaginalis are not only responsible for HIV propagation but also for the transmission of many other diseases such as cancer. We extensively re-read the literature and accordingly have revised the introduction section as per the reviewer’s recommendation. In addition, we provided specific literature pertaining to developing nations. The unmarked manuscript contains updated lines 49-111, while the previous edition included lines 55–87.

**Materials and Methods**

Comment 2: I am not sure when the MS was submitted. The latest BDHS data sets conducted in 2017-18 are released by the end of November or in early of December, 2020. If so, why the authors have not used most recent data?

Response: Thank you for highlighting this. At the time of submission (December 2020, https://dhsprogram.com/data/available-datasets.cfm), the BDHS 2017-18 data set was available. However, we were unable to find any data on STIs in the 2017-18 BDHS. Although the variables exist in the dataset, we did not find any observations for them.

Comment 3: A flow chart of selection of the 16,858 from 17,886 women may improve the quality of the MS.

Response: Thank you for your suggestion. We have included a flow chart in the TIFF file, titled "A flow chart of study population selection from the Bangladesh demographic health survey (BDHS) 2014."

Comment 4: Since the data have been gathered through a multi-stage procedure, why did not the authors use multilevel logistic regression analysis. The empirical studies suggest that women’s empowerment are associated with clusters. I would suggest employing multilevel logistic regression analysis to quantify the unobserved effects on having any STI among married women captured by clusters.

Response: We would like to thank the reviewer for raising this crucial suggestion regarding employing the multilevel logistic regression model. We previously considered the marginal model and adjusted the weight adopted from BDHS in our analysis. However, we have now re-run our analysis using multilevel mixed-effect logistic regression. Moreover, we have revised the statistical analysis section (lines 155-184 in the new edition), the results section (lines 213-240 in the new edition), and table-4 to reflect these changes. In addition, we have updated our discussion section in line with the results section.

Comment 5: How the missing values were managed by stata?

Response: Thank you for highlighting this issue. We did not employ any statistical analysis process such as missing at random or missing completely at random to manage missing values in our analysis. We conducted the analysis using the available complete data in the sample. In the BDHS report the authors did not conduct any missing value operation rather they reported if the missing value were presents. We have added a footnote to Table-3 to indicate that the total may not equal 100.0 percent due to missing values.
