# Supplementary material for: Women empowerment and sexually transmitted infections: Evidence from Bangladesh demographic and health survey 2014
Source: PLoS One. 2022 Feb 16;17(2):e0263958. doi: 10.1371/journal.pone.0263958 (PMC8849524; doi:10.1371/journal.pone.0263958)
Supplement: Attachment — Submitted filename: Responses to Reviewers.docx [file pone.0263958.s002.docx]

**Response to Journal Requirements**

Please review your reference list to ensure that it is complete and correct. If you have cited papers that have been retracted, please include the rationale for doing so in the manuscript text, or remove these references and replace them with relevant current references. Any changes to the reference list should be mentioned in the rebuttal letter that accompanies your revised manuscript. If you need to cite a retracted article, indicate the article’s retracted status in the References list and also include a citation and full reference for the retraction notice.

**Response:** Thank you very much. It is really appreciated. We modified certain references during the Round 1 revision. The following is a list of changed references.

**Retracted references (lines 520-642 in track changed version)**

1. Fabrizio S, Kolovich L, Newiak M, Agarwal A, Yin RJ. Pursuing Women's Economic Empowerment. (IMF Web site): Accessed July 13, 2019. https://www.imf.org/en/Publications/Policy-Papers/Issues/2018/05/31/pp053118pursuing-womens-economic-empowerment. , 2019.

2. Gibson CJ, Huang AJ, McCaw B, Subak LL, Thom DH, Van Den Eeden SK. Associations of Intimate Partner Violence, Sexual Assault, and Posttraumatic Stress Disorder With Menopause Symptoms Among Midlife and Older Women. JAMA Intern Med. 2019;179(1):80-7. Epub 2018/11/20. doi: 10.1001/jamainternmed.2018.5233. PubMed PMID: 30453319.

3. Treves-Kagan S, El Ayadi AM, Morris JL, Graham LM, Grignon JS, Ntswane L, et al. Sexual and Physical Violence in Childhood Is Associated With Adult Intimate Partner Violence and Nonpartner Sexual Violence in a Representative Sample of Rural South African Men and Women. J Interpers Violence. 2019:886260519827661. Epub 2019/02/09. doi: 10.1177/0886260519827661. PubMed PMID: 30735091

5. Prakash R, Singh A, Pathak PK, Parasuraman S. Early marriage, poor reproductive health status of mother and child well-being in India. J Fam Plann Reprod Health Care. 2011;37(3):136-45. Epub 2011/06/02. doi: 10.1136/jfprhc-2011-0080. PubMed PMID: 21628349

6. Kaljee LM, Green M, Riel R, Lerdboon P, Tho le H, Thoa le TK, et al. Sexual stigma, sexual behaviors, and abstinence among Vietnamese adolescents: implications for risk and protective behaviors for HIV, sexually transmitted infections, and unwanted pregnancy. J Assoc Nurses AIDS Care. 2007;18(2):48-59. Epub 2007/04/04. doi: 10.1016/j.jana.2007.01.003. PubMed PMID: 17403496; PubMed Central PMCID: PMCPmc2063998

9. Ilankoon IMPS, Goonewardena CSE, Perera PPR, Fernandopulle R. Vaginal Discharge Women's Health Seeking Behaviours And Cultural Practices. 2015.

10. Gomes CMM, Giraldo PC, Gomes FdAM, Amaral R, Passos MRL, Gonçalves AKdS. Genital ulcers in women: clinical, microbiologic and histopathologic characteristics. Brazilian Journal of Infectious Diseases. Apr. 2007;11(2).

11. Sheffield JS, Wendel GD, Jr., McIntire DD, Norgard MV. Effect of genital ulcer disease on HIV-1 coreceptor expression in the female genital tract. The Journal of infectious diseases. 2007;196(10):1509-16. Epub 2007/11/17. doi: 10.1086/522518. PubMed PMID: 18008231.

24. Hebling EM, Guimaraes IR. Women and AIDS: gender relations and condom use with steady partners. Cad Saude Publica. 2004;20(5):1211-8. Epub 2004/10/16. doi: /S0102-311x2004000500014. PubMed PMID: 15486663.

25. MacPherson EE, Sadalaki J, Njoloma M, Nyongopa V, Nkhwazi L, Mwapasa V, et al. Transactional sex and HIV: understanding the gendered structural drivers of HIV in fishing communities in Southern Malawi. J Int AIDS Soc. 2012;15 Suppl 1:1-9. Epub 2012/06/22. doi: 10.7448/ias.15.3.17364. PubMed PMID: 22713352; PubMed Central PMCID: PMCPmc3499929.

26. Bloom SS, Wypij D, Gupta MD. Dimensions of women’s autonomy and the influence on maternal health care utilization in a north Indian city. Demography. 2001;38(1):67-78.

33. Haque SE, Rahman M, Mostofa MG, Zahan MS. Reproductive health care utilization among young mothers in Bangladesh: does autonomy matter? Women's Health Issues. 2012;22(2):e171-e80

**Added references (lines 654-807 in track changed version)**

1. Korenromp EL, Rowley J, Alonso M, Mello MB, Wijesooriya NS, Mahiane SG, et al. Global burden of maternal and congenital syphilis and associated adverse birth outcomes-Estimates for 2016 and progress since 2012. PLoS One. 2019;14(2):e0211720. Epub 2019/02/28. doi: 10.1371/journal.pone.0211720. PubMed PMID: 30811406; PubMed Central PMCID: PMCPMC6392238.

2. Prevention CfDCa. Syphilis - CDC Fact Sheet. Available from: <https://www.cdc.gov/std/syphilis/stdfact-syphilis.htm>.

4. Organisation WH. Sexually transmitted infections (STIs) February 28 2019. Available from: <https://www.who.int/news-room/fact-sheets/detail/sexually-transmitted-infections-(stis)>.

5. Wand H, Ramjee G. Assessing and evaluating the combined impact of behavioural and biological risk factors for HIV seroconversion in a cohort of South African women. AIDS care. 2012;24(9):1155-62.

6. Davey DJ, Shull H, Billings J, Wang D, Adachi K, Klausner J. Prevalence of curable sexually transmitted infections in pregnant women in low-and middle-income countries from 2010 to 2015: a systematic review. Sexually transmitted diseases. 2016;43(7):450.

7. Chesson HW, Mayaud P, Aral SO. Sexually transmitted infections: impact and cost-effectiveness of prevention. 2017.

8. Mayaud P, Mabey D. Approaches to the control of sexually transmitted infections in developing countries: old problems and modern challenges. Sexually transmitted infections. 2004;80(3):174-82.

9. Alomair N, Alageel S, Davies N, Bailey JV. Sexually transmitted infection knowledge and attitudes among Muslim women worldwide: a systematic review. Sexual and reproductive health matters. 2020;28(1):1731296.

10. Jahangir YT, Arora A, Liamputtong P, Nabi MH, Meyer SB. Provider Perspectives on Sexual Health Services Used by Bangladeshi Women with mHealth Digital Approach: A Qualitative Study. International Journal of Environmental Research and Public Health. 2020;17(17):6195

14. Kabir A, Rashid MM, Hossain K, Khan A, Sikder SS, Gidding HF. Women’s empowerment is associated with maternal nutrition and low birth weight: Evidence from Bangladesh Demographic Health Survey. BMC women's health. 2020;20:1-12

15.James-Hawkins L, Peters C, VanderEnde K, Bardin L, Yount KM. Women’s agency and its relationship to current contraceptive use in lower-and middle-income countries: A systematic review of the literature. Global Public Health. 2018;13(7):843-58

16. Ahmed S, Creanga AA, Gillespie DG, Tsui AO. Economic status, education and empowerment: implications for maternal health service utilisation in developing countries. PloS one. 2010;5(6):e11190

18. Yaya S, Uthman OA, Ekholuenetale M, Bishwajit G. Women empowerment as an enabling factor of contraceptive use in sub-Saharan Africa: a multilevel analysis of cross-sectional surveys of 32 countries. Reproductive health. 2018;15(1):1-12

19. Afroja S, Rahman M, Islam L. Women’s Autonomy and Reproductive Healthcare-Seeking Behavior in Bangladesh: Further Analysis of the 2014 Bangladesh Demographic and Health Survey.

20. Story WT, Burgard SA. Couples’ reports of household decision-making and the utilisation of maternal health services in Bangladesh. Social science & medicine. 2012;75(12):2403-11.

21. Parvin GA, Ahsan SR, Chowdhury MR. Women empowerment performance of income generating activities supported by Rural Women Employment Creation Project (RWECP): A case study in Dumuria Thana, Bangladesh. The Journal of Geo-Environment. 2004;4(1):47-62.

22. Schuler SR, Lenzi R, Badal SH, Bates LM. Women’s empowerment as a protective factor against intimate partner violence in Bangladesh: a qualitative exploration of the process and limitations of its influence. Violence against women. 2017;23(9):1100-21.

23. Boroumandfar Z, Kianpour M, Zargham A, Abdoli S, Tayeri K, Salehi M, et al. Changing beliefs and behaviors related to sexually transmitted diseases in vulnerable women: A qualitative study. Iranian journal of nursing and midwifery research. 2017;22(4):303.

24. Alam N, Streatfield PK, Khan SI, Momtaz D, Kristensen S, Vermund SH. Factors associated with partner referral among patients with sexually transmitted infections in Bangladesh. Social science & medicine. 2010;71(11):1921-6.

25. World Economic Forum, Global Gender Gap Report 2021, Insight Report. Geneva, Switzerland: World Economic Forum, 2021

26. Organization IL. Labor force participation rate, female (% of female population ages 15+) ( modeled ILO estimate)- Bangladesh January 29, 2021. Available from: <https://data.worldbank.org/indicator/SL.TLF.CACT.FE.ZS?locations=BD>

27. Osborn D, Cutter A, Ullah F. Universal sustainable development goals. Understanding the Transformational Challenge for Developed Countries. 2015

34. Hernán MA, Hernández-Díaz S, Werler MM, Mitchell AA. Causal knowledge as a prerequisite for confounding evaluation: an application to birth defects epidemiology. American journal of epidemiology. 2002;155(2):176-84.

35. Victora CG, Huttly SR, Fuchs SC, Olinto M. The role of conceptual frameworks in epidemiological analysis: a hierarchical approach. International journal of epidemiology. 1997;26(1):224-7.

43. Haque SE, Rahman M, Mostofa MG, Zahan MS. Reproductive health care utilisation among young mothers in Bangladesh: does autonomy matter? Women's Health Issues. 2012;22(2):e171-e80.

51. Adriel Monkam Tchokossa RN B, Timothy Golfa RN M, Omowumi Romoke Salau RN M, FWACN AAO. Perceptions and experiences of intimate partner violence among women in Ile-Ife Osun state Nigeria. International Journal of Caring Sciences. 2018;11(1):267-78

52. Payton E, Eluka N, Brown R, Dudley WN. Women's perceptions of intimate partner violence in Zambia. Violence and gender. 2019;6(4):219-26.

53. Sanawar SB, Islam MA, Majumder S, Misu F. Women’s empowerment and intimate partner violence in Bangladesh: investigating the complex relationship. Journal of biosocial science. 2019;51(2):188-202.

54.Jesmin SS. Married women’s justification of intimate partner violence in Bangladesh: Examining community norm and individual-level risk factors. Violence and victims. 2015;30(6):984-1003.

55. Silverman JG, Gupta J, Decker MR, Kapur N, Raj A. Intimate partner violence and unwanted pregnancy, miscarriage, induced abortion, and stillbirth among a national sample of Bangladeshi women. BJOG: An International Journal of Obstetrics & Gynaecology. 2007;114(10):1246-52.

**Response to Reviewer Comments**

1. Line 34 lacks and between cross-tab and logistic regression

Response: Thank you. We have already amended it in the Round 1 revision. We have removed the “ cross-tab, logistic regression” (line 34 in track changed version). We have now included “cross-tabulation, the conceptual framework technique and multivariable multilevel mixed-effect logistics regression” ( line 38 in track changed version; line 30 in clean version)

2. Line 37, express figures in the same format. I see that some of them presented to one decimal place yet others are presented to two decimal places. Make this consistent throughout the manuscript

Response: Thank you for highlighting this important concern. We appreciate it. We have presented numbers in a similar format. (lines 47-53 in track change version; lines 33-39 in clean version)

3. Line 57 should be women’s well-being

Response: Thank you. We appreciate it, but we have already revised our introduction in Round 1 (lines 81-97, 107-115,124-144, 151-161,167-171 included in Round 1 ). We have already removed “women well-being” ( line 78 track changed version) and revised 1^st^ paragraph ( lines 81-97 in track changed version; lines 49-65 in cleaned version).

4. Line 62 : reported to lead to

Response: We are grateful for your consideration. However, our introduction was previously revised in Round 1(lines 81-97, 107-115,124-144, 151-161,167-171 included in Round 1). We've previously removed “line 62” ( line 100 track changed version) and included revised sentences (lines 107 to 115 in track changed version; lines 66-74 in cleaned version).

5. Line 105: Outcome variables

Response: Many thanks for your comment. We have amended the statement (line 196 in track changed version; line 135 in cleaned version )

6. In table 1, I think you refer to recoded not recorded

Response: We appreciate your comment. In table 1, now we have changed “recored” to “recoded”.

7. In table 3, control and decision making variables are not presented well. The names of the categories are cut – some words are below the lines. I think you can consider having the table in a smaller font so that the names of the variables and their categories appear in whole.

Response: We appreciate this concern. We have corrected it (both in track changed version & cleaned version)

8. In table 4, the word is reference not referent

Response: We appreciate this concern. But we have already updated table 4 according to the Round 1 revision. We also changed “referent” to ”reference” ( Table 4 both in track changed version & clean version). We have also format Table 3 and Table 4 ( both in track changed version & clean version )

9. Line 213, use have instead of has

Response: Thank you for the suggestion. We have already updated line 213 according to the Round 1 revision (lines 375-385,422-444,447-455,473-477 included in Round 1). We have now rewritten it as “ In this study, 5.59% and 10.84% of the respondents reported genital sores and abnormal genital discharge, respectively.” ( line 375-376 in track changed version; lines 252-253 in cleaned version)

10. Line 214, in not from

Response: Thank you for the suggestion. We have already modified line 214 according to the Round 1 revision (lines 375-385,422-444,447-455,473-477 included in Round 1). We have now rewritten it as “ Women who actively participated in joint decision-making with their husbands or partners regarding their own and child's health were significantly less likely to report STI symptoms.” ( line 378-379 in track changed version; lines 255-256 in cleaned version)

11. Line 239, delete help

Response: Thank you for the suggestion. We have already modified line 239 according to the Round 1 revision (lines 375-385,422-444,447-455,473-477 included in Round 1). We have now rewritten it as “ Receiving support from the husband benefits women in terms of better resource utilisation and the uptake of necessary health services” ( line 391-392 in track changed version; lines 267-268 in cleaned version)

12. You need a final English edit for the manuscript

Response: Thank you. We have adequately corrected the English mistake.
