# Supplementary material for: Women empowerment and sexually transmitted infections: Evidence from Bangladesh demographic and health survey 2014
Source: PLoS One. 2022 Feb 16;17(2):e0263958. doi: 10.1371/journal.pone.0263958 (PMC8849524; doi:10.1371/journal.pone.0263958)
Supplement: Attachment — Submitted filename: Responses to Reviewers.docx [file pone.0263958.s003.docx]

Comment 1 : Congratulations for reaching this far.
The manuscript needs more editing for typographical errors. These are not major but need to be checked so that it reads better. Specifically, some sentences lack joining words, do not read well and the tenses are not correct, which I believe the authors can sort. For example the first sentence in the Statistical analysis section

**Response:** We are grateful for your consideration. As per your feedback, we have eliminated the typographical errors and performed a thorough line-by-line edit of the manuscript to facilitate better expression.

Comment 2 : I realize that you use STI and STIs as an acronym for sexually transmitted infections interchangeably, I suggest you use one for consistency.

Thank you for your suggestion. Now we have used the term STIs consistently in the manuscript. We have also described STI as STIs and also written symptoms of STIs in line 69, 73, 77, 78, 84, 112, 123, 126, 130, 136, 163, 180, 279, 282, 284, 349, 350, 353,355, 365,367, 372, 374,382.

Comment3 : Additionally, LMIC/LMICs/LMIC countries are used interchangeably. There is need to check them for consistency

**Response:** We appreciate your comment. We have replaced LMICs as LMIC in line 69, line 97.

3. In the Abstract, What does “conceptual framework technique” mean

**Response:** Many thanks. Conceptual frameworks assist the use of multivariate approaches by managing complicated hierarchical interrelationships between variables in light of social and biological knowledge. The conceptual framework depicts causal pathways between the outcome and explanatory variables based on empirical knowledge from previous study findings and the researchers’ experience. The factors included in the multivariable model have been chosen using this causal / hierarchical relationship. However, it seems that the word “technique” has added some confusion. That is why, in the manuscript, we have just included “conceptual framework” instead of “conceptual framework technique” (lines 157- 168).

35.Victora CG, Huttly SR, Fuchs SC, Olinto M. The role of conceptual frameworks in epidemiological analysis: a hierarchical approach. International journal of epidemiology. 1997;26(1):224-7.

4. Line 55, begin the sentence with A instead of In, so the sentence should read “A systematic review of studies in 30 low- and middle-income countries (LMIC) showed that STI among women remain ……”

**Response:** Thank you for highlighting the issues. We changed it in Round 1, and it is currently written in the form you suggested. That is why it has remained unchanged in this version.

5. In table 2 change “Women participate in a variety of decision-making processes” to “Women’s participation in decision-making”

**Response:** Thank you. We have changed “Women participate in a variety of decision-making processes” to “Women’s participation in decision-making” in Table 2, Table 3, Table 4.
